# Diagnostic role of new Doppler index in assessment of renal artery stenosis

**DOI:** 10.1186/1476-7120-4-4

**Published:** 2006-01-25

**Authors:** Sergio Chain, Hector Luciardi, Gabriela Feldman, Sofia Berman, Ramón N Herrera, Javier Ochoa, Juan Muntaner, Eduardo M Escudero, Ricardo Ronderos

**Affiliations:** 1 Department of Cardiac and Vascular Ultrasonography, Centro Modelo de Cardiología and Centro Radiológico Mendez Collado, Universidad Nacional de Tucumán, San Miguel de Tucumán, Argentina; 2 Muñecas 444, (T4000IKJ) San Miguel de Tucumán, Argentina; 3 Magister in Cardiovascular Ultrasound, Universidad Nacional de La Plata, Argentina; 4 60 and 120 streets, (1900) La Plata, Argentina

## Abstract

**Background:**

Renal artery stenosis (RAS) is one of the main causes of secondary systemic arterial hypertension. Several non-invasive diagnostic methods for RAS have been used in hypertensive patients, such as color Doppler ultrasound (US). The aim of this study was to assess the sensitivity and specificity of a new renal Doppler US direct-method parameter: the renal-renal ratio (RRR), and compare with the sensitivity and specificity of direct-method conventional parameters: renal peak systolic velocity (RPSV) and renal aortic ratio (RAR), for the diagnosis of severe RAS.

**Methods:**

Our study group included 34 patients with severe arterial hypertension (21 males and 13 females), mean age 54 (± 8.92) years old consecutively evaluated by renal color Doppler ultrasound (US) for significant RAS diagnosis. All of them underwent digital subtraction arteriography (DSA). RAS was significant if a diameter reduction > 50% was found. The parameters measured were: RPSV, RAR and RRR. The RRR was defined as the ratio between RPSV at the proximal or mid segment of the renal artery and RPSV measured at the distal segment of the renal artery. The sensitivity and specificity cutoff for the new RRR was calculated and compared with the sensitivity and specificity of RPSV and RAR.

**Results:**

The accuracy of the direct method parameters for significant RAS were: RPSV >200 cm/s with 97% sensitivity, 72% specificity, 81% positive predictive value and 95% negative predictive value; RAR >3 with 77% sensitivity, 90% specificity, 90% positive predictive value and 76% negative predictive value. The optimal sensitivity and specificity cutoff for the new RRR was >2.7 with 97% sensitivity (p < 0.004) and 96% specificity (p < 0.02), with 97% positive predictive value and 97% negative predictive value.

**Conclusion:**

The new RRR has improved specificity compared with the direct method conventional parameters (RPSV >200cm/s and RAR >3). Both RRR and RPSV show better sensitivity than RAR for the RAS diagnosis.

## Background

Renovascular hypertension might account for 1–5% of all cases of hypertension [1,2]. However, it affects 15–30% of patients who have clinical criteria suggestive of renovascular hypertension as refractory hypertension to an appropiate three-drug treatment associated either with moderate impairment of renal function or with carotid, peripheral or coronary atherosclerotic disease. The renovascular hypertension term focuses on the causal relationship between RAS and its clinical consequences (hypertension and renal failure) [3]. Atherosclerosis accounts for 90% of cases of RAS, and usually involves the ostium and the proximal segment of the main renal artery and the perirenal aorta. Fibromuscular dysplasia accounts for less than 10% of cases of RAS. Fibromuscular dysplasia tends to affect girls and women who are between 15 and 50 years of age, and frequently involves the distal two segments of the renal artery and its branches [3]. Atherosclerotic RAS is a common and progressive disease. Its prevalence increases with age, particularly in patients who have diabetes, aortoiliac occlusive disease, coronary artery disease or hypertension [4]. Owing to improvements in the techniques used for screening, it is now recognized that 40–50% of patients with occlusive disease of the lower limb and 15–30% of patients with coronary artery disease have recognizable RAS [5,6]. Reports of end-stage renal disease indicate a prevalence of RAS of 10–22% [7,8].

A diagnosis of atherosclerotic RAS can be suspected on the clinical grounds mentioned, but can only be established through specific diagnostic procedures [4,9,10]. Because renovascular hypertension could by treated by percutaneous transluminal angioplasty, endovascular stent placement or surgical revascularization, several non-invasive methods have been advocated to screen for suspected renovascular disease [11,12]. Renal color Doppler ultrasound (US) has been proposed as an effective modality for the diagnosis of RAS. The use of Doppler US in patients with hypertension has led to an increase in the diagnosis of RAS [13,14].

The direct method Doppler parameters evaluate the renal artery peak systolic velocity (RPSV) and the aortic peak systolic velocity to determine the maximal RPSV and the renal aortic ratio (RAR). The new direct method Doppler parameter, the renal renal ratio (RRR), was defined as the rate between RPSV at the proximal or mid segment of the renal artery and RPSV measured at the distal segment of the renal artery.

The indirect method Doppler parameters evaluate the post stenostic "tardus-parvus" phenomenon in the intrarenal arterial Doppler waveforms [14]. We did not evaluate this indirect method parameters in this research.

The aim of this study was to assess the sensitivity and specificity of a new renal Doppler US direct method parameter, the renal renal ratio (RRR), and to compare it with the sensitivity and specificity of another direct method conventional parameters, renal peak systolic velocity (RPSV) and renal aortic ratio (RAR), for the diagnosis of severe RAS.

## Methods

Between October 2001 and October 2003, 34 consecutive patients who had severe hypertension and more than three of clinical features suggestive of RAS (Table 1) were referred to the Department of Cardiac and Vascular Ultrasonography at our institutions and were prospectively evaluated by renal color Doppler US. Patient characteristics are summarized in Table 2. We have made a comparative study of three Doppler US parameters: RPSV, RAR, and the new RRR for the diagnosis of RAS. All patients underwent digital subtraction arteriography (DSA), as the "gold standard", within 4 weeks of color Doppler US. The reading of the DSA and the renal color Doppler US was blinded from one another.

**Table 1:** Clinical features suggestive of renal artery stenosis

Epigastric or flank bruit (systolic or diastolic) Accelerated or malignant hypertension Unilateral small kidney discovered with any clinical study Severe hypertension in a child or young adult or aged more than 50 years Sudden development or worsening of hypertension at any age Hypertension and unexplained impairment of renal function Sudden worsening of renal function in a hypertensive patient Hypertension refractory to an appropriate three-drug regimen Impairment of renal function after treatment with an ACE inhibitor Hypertension and extensive arterial occlusive disease (peripheral vascular and coronary arterial disease)
ACE: angiotensin-converting enzyme
Three signs were found in 11 patients, 4 signs in 10 and ≥5 signs in 13 patients

**Table 2:** Demographic characteristics of the patients.

		N = 34	%
Sex	Women	13	33
Age		54 (± 8.92)	-
Hypertension		34	100
Dyslipidemia		14	40
Smoking status		7	21
Diabetes		6	19
BMI (mean)		30 kg/m^2 ^(+/-5)	-

BMI, body mass index

**Figure 1:**
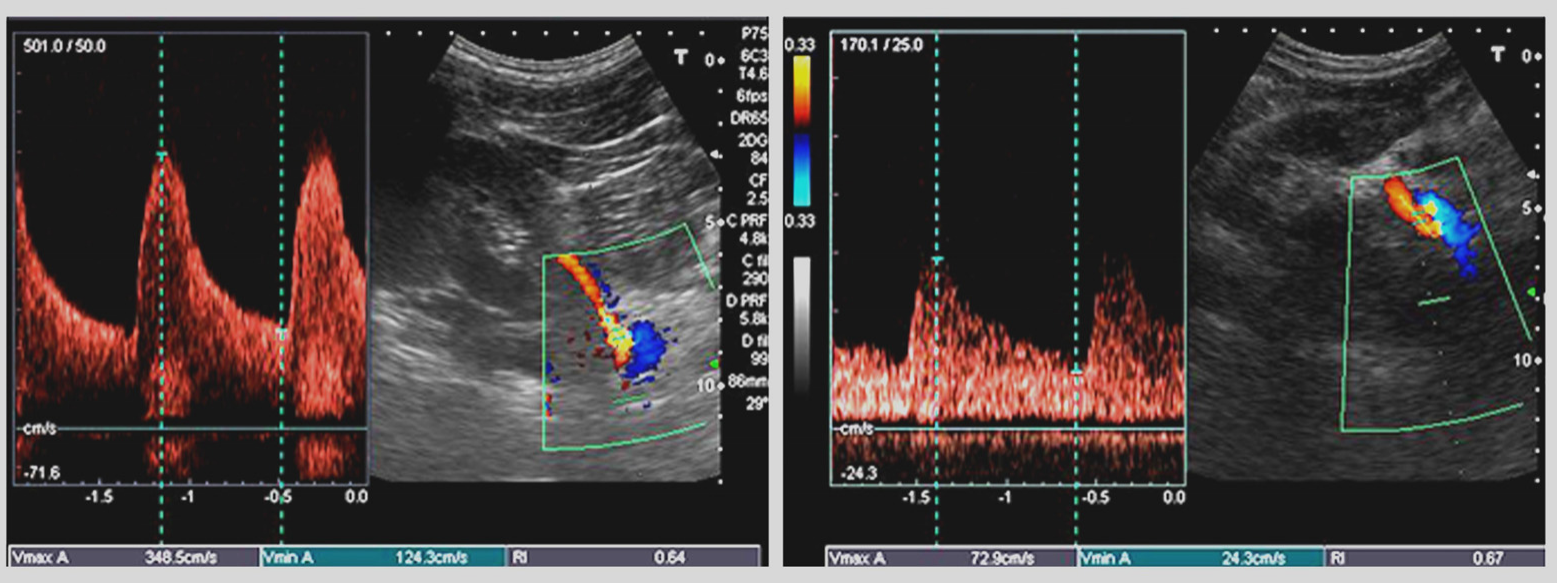
**Renal renal ratio at right renal artery stenosis**. Panel a. Renal peak systolic velocity at the proximal segment of the right renal artery: 348 cm/s. Panel b. Renal peak systolic velocity at the distal renal artery segment : 72.9 cm/s. Renal-renal ratio = 348 cm/s / 73 cm/s = 4.76 (significant renal artery stenosis > 2.7).

The conventional direct method Doppler US parameters evaluated were RPSV and RAR (that is, RPSV divided by the peak systolic velocity at the abdominal aorta). In an attempt to improve the detection of RAS, a new direct method Doppler parameter, RRR, was also evaluated. The RRR was defined as the RPSV at the proximal or mid renal artery segment divided by the RPSV at the distal renal artery segment (Figure. 1a,b. and 2).

**Figure 2:**
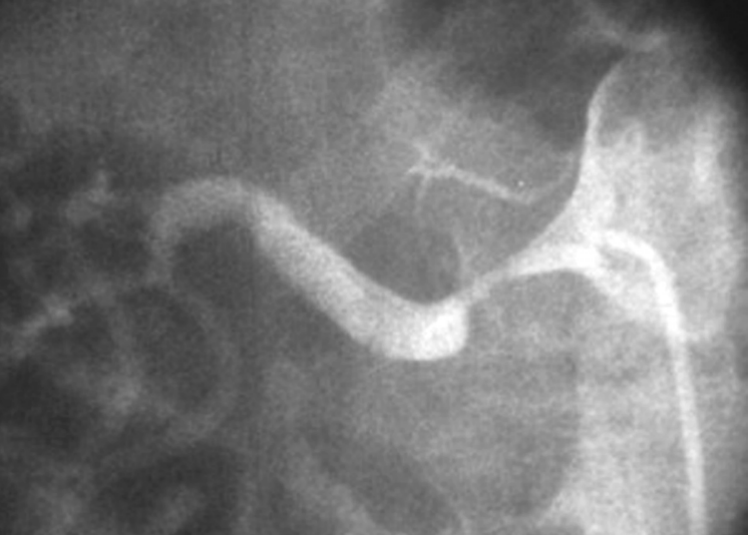
Anteroposterior digital subtraction arteriography shows a severe right renal artery stenosis.

RRR = RPSV (proximal or mid renal artery)/RPSV (distal renal artery)

We have only evaluated the main renal arteries, we did not use the RRR to analize the accessory arteries.

### Imaging technique

#### Ultrasound imaging

Renal Doppler US was performed in our departments using a Toshiba Nemio 30 machine with low-frequency curve transducers (3–6 MHz) and low-frequency sectorial transducers (2–4 MHz) to allow greater penetration of the US beam.

All examinations were performed by a single physician with more than five-years experience in vascular sonography. The examinations were performed in the morning if possible and the patients were recommended to observe a 10-hour fasting period beforehand. The complete procedure was generally completed within approximately 30–35 minutes. All examinations were started with the patients in the supine position, in order to visualize the abdominal aorta and the origin and proximal course of the main renal artery. The patients were then kept recumbent in various different positions: the left decubitus position to explore the right renal artery, the ventral decubitus position to explore the left renal artery or the right decubitus position in a few cases.

The maximum diameter and the peak systolic velocity in the abdominal aorta were obtained in a longitudinal section of its proximal segment.

Epigastric transverse scans allowed us to identify the main renal arteries, which originate laterally (anterolaterally for the right artery and posterolaterally for the left artery) in the abdominal aorta about 1 cm under the emergence of the superior mesenteric artery. The right and left renal arteries also have a proximal course just posterior to the left renal vein under normal conditions. Therefore, the origin and the proximal segment of the renal arteries were initially assessed in a transverse section of the abdominal aorta in the epigastric region. Every patient had all segments of bilateral renal arteries interrogated: proximal, mid and distal. Depending on the anatomy of the patient, almost the entire length of the renal arteries could be assessed.

We performed direct imaging of the main renal artery with the color window and pulsed wave (PW) Doppler. When the renal artery image showed homogeneous color and standard velocity, we diagnosed normal renal artery patency.

For the diagnosis of RAS using the conventional technique, we performed direct imaging of the main renal artery. Color imaging revealed the presence of stenosis through signs of turbulence in the systolic phase, which was generally yellow and green. Subsequently, the PW Doppler was positioned in the area of interest in the center of the vessel, at the site identified by increased flow velocities and turbulence. The gain, filter and scales of the velocity curves of the PW Doppler were adjusted to provide an adequate curve for measuring the velocities. When the renal artery PW Doppler showed increased peak systolic velocity, we suspected RAS. The highest value of the three measurements of RPSV performed from different views was chosen in each case.

For the abdominal aorta and main renal arteries, the angle of incidence of the pulsed Doppler was maintained less than 60°, which was essential in order to obtain accurate measurements. We used the same or very similar angle correction at proximal, mid and distal segments of the renal artery. It was a very important technical detail. The velocities at proximal, mid and distal segments of the renal artery were assessed using obliqual sections.

Doppler samples were taken in apnoea, when the image of the artery was found to be optimal in the respiratory cycle.

We did not use contrast echo in case of difficult renal artery imaging.

#### Digital substraction angiography

Digital subtraction angiography was performed in all patients. This was performed using a 5-F pigtail catheter, with the tip positioned through the right or left femoral artery just proximal to the renal arteries. A non-ionic contrast material was injected and the images were obtained in the anteroposterior, left anterior oblique and right anterior oblique projections. The criterion for anatomically significant RAS at angiography was 50% or greater renal artery narrowing. The stenosis was assesed by calipers.

### Statistical analysis

The sensitivity, specificity, positive predictive value and negative predictive value for the detection of significant RAS were calculated independently for the three parameters: RPSV, RAR and RRR, and its sensitivity and specificity were compared. The sensitivity for detecting stenosis was calculated as the number of true-positive findings according to color Doppler US divided by the number of positive findings by DSA. The specificity was calculated as the number of true-negative findings according to color Doppler US divided by the number of negative findings by DSA.

Additionally, receiver operating characteristic (ROC) curves were computed to compare the parameters, which provided a graphic description of the performance of the tested variables towards RAS detectability. The curves were generated from data obtained through sensitivity/specificity analysis of the variables.

The chi square test was used to evaluate the difference among the three direct method color Doppler US parameters. All statistical tests were two-tailed and performed at the 0.05 level of significance. The confidence intervals were two-sided with 95% intervals. The SPSS 10.0 statistical package was used for all the calculations. The protocol was approved by our institutional ethics committee and all the patients provided written informed consent.

## Results

Ninety seven percent of our patients were successfully examined with color Doppler US, and the results were good enough to be included in the analysis in all cases. The intra-examiner variability was good (correlation 0.86, coefficient of variation 8.9%). The sensitivity, specificity and predictive values of the direct method renal Doppler parameters were assessed in 34 patients. We found with DSA 35 main stenotic renal arteries and 29 main normal renal arteries (Table 3).

**Table 3:** DSA results

	N° of pts	RA without stenosis	RA with stenosis	RA with total oclusion
Pts without RAS	5	10	0	0
Pts with unilateral RAS	23	19	23	4
Pts with bilateral RAS	6	0	12	0

Total	34	29	35	4

DSA: Digital substraction angiography; pts.: patients, RAS: renal-artery stenosis, RA: renal arteries.

### Sensitivity and specificity of the new RRR

The best estimated cutoff value for the new RRR was 2.7, as defined by the intersection of the sensitivity/specificity curves at the maximum values (Table 4).

**Table 4:** Cutoff values for RRR sensitivity and specificity

Cutoff	Sensitivity (%)	Specificity (%)	PPV (%)	NPV (%)
2.9	89	100	100	88
2.8	94	100	100	93
**2.7**	**97**	**96**	**97**	**96**
2.6	97	93	95	96
2.5	100	86	89	100

Cutoff: cutoff value; RRR: renal-renal ratio; PPV: positive predictive value; NPV: negative predictive value

### Sensitivity and specificity of the conventional direct method parameters

The sensitivity and specificity of the conventional direct method parameters (RPSV and RAR) were evaluated. We found that RPSV >200 cm/s and RAR >3 were indicative of severe RAS as it is referred in the literature [14]. RPSV >200 cm/s resulted in a sensitivity of 97%, a specificity of 72%, a positive predictive value of 81% and a negative predictive value of 95% in terms of the diagnostic accuracy for RAS. A severe RAS diagnosis with RAR >3 yielded a sensitivity of 77%, a specificity of 90%, a positive predictive value of 90% and a negative predictive value of 76%.

Additionally, the RRR sensitivity (97%) and specificity (96%) values were compared with the other mentioned direct-method parameters (RPSV >200 cm/s and RAR >3) (Table 5). The sensitivities of RRR and RPSV were highest than RAR for RAS diagnosis (p < 0.004). The specificity of RRR was statistically superior compared with RPSV and RAR (p < 0.02).

**Table 5:** Sensitivity and specificity of the direct parameters

Parameter	Sensitivity (%)	Specificity (%)	PPV (%)	NPV (%)
RPSV > 200 cm/s	97	72	81	95
RAR > 3	77	90	90	76
RRR > 2.7	97	96	97	97

RPSV: renal peak systolic velocity; RAR: renal/aortic ratio; RRR: renal-renal ratio PPV: positive predictive value; NPV: negative predictive value

**Figure 3:**
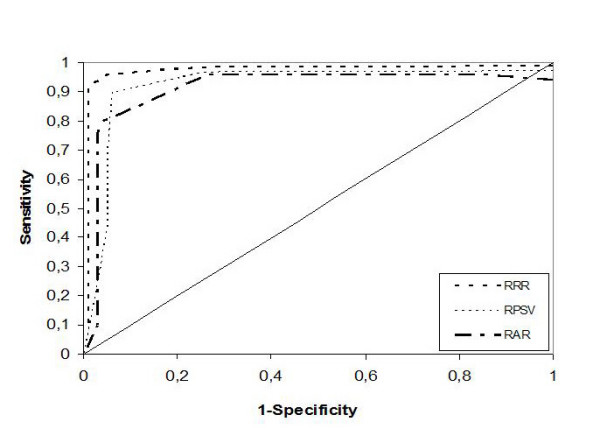
**ROC curve of renal renal ratio**. The area under the curve was greater for RRR (0.99) than for the other tested parameters: RPSV (0.95) and RAR (0.93).

The ROC curve analysis showed that the area under the curve for RRR(0.99) was greater than that for both RPSV and RAR (0.95 and 0.93 respectively) (Fig. 3).

## Discussion

The discriminatory capacities of the direct method parameters applied with color Doppler US were evaluated based on their statistical significance, as well as on the results of the sensitivity and specificity analysis.

### Sensitivity and specificity of the direct method parameters

Hoffman et al. used an RPSV >180 cm/s to discriminate RAS with a sensitivity of 95% and a specificity of 90% [16]. Souza de Oliveira et al. showed a sensitivity of 83.3%, a specificity of 89.5% and a cutoff value of approximately 150 cm/s [14]. In our population, the best cutoff value was 200 cm/s with a sensitivity of 97% and a specificity of 72%.

Hoffman et al. using an RAR of 3.5 identified a RAS >60% with a sensitivity of 92% and a specificity of 62% [16]. Similar values of sensitivity (ranging from 92 to 95%) and higher specificity results (ranging from 88 to 90%) were obtained by others authors, such as Rabbia et al. and Conkbayir et al. [17,18]. In Souza de Oliveira's study population, the best cutoff value for this index was considered to be approximately 1.8, which was much less than the 3.5 cutoff value, and showed satisfactory results for sensitivity (83.3%) and a low specificity (78.9%) [14]. In our study population, the best cutoff value was 3, with a sensibility of 77% and a specificity of 90%. These variable results are probably related to differences in the estimated cutoff values and the different angiographic degrees of stenosis (ranging from 50 to 70%) that were considered to be significant by some previous authors [14,16-18].

### The new RRR

Our new proposed diagnostic index, RRR, is based on the fact that increased blood flow velocity across the stenosis and the immediate post-stenotic segments, and the observed decrease in blood flow velocity distal to the stenosis is proportional to the degree of stenosis [14,19].

In our current study, the RRR was particularly usefull because produced specificity results for RAS diagnosis that were superior to those of RPSV. RRR uses comparative velocity and it increases the diagnostic assurance to the operator. An advantage of our proposed index over RAR was that the diameter of the main renal artery in the distal segment was similar to those of the proximal and middle segments; by contrast, RAR uses the abdominal aorta, which has a much larger diameter. This enables a better comparison of the flow velocity between proximal and distal segments [20].

The most commonly used indirect method parameters are the systolic acceleration ratio, the acceleration time, and the intraparenchymal resistance ratio difference between the right and left kidneys [14,21]. We did not use these indirect method parameters in our research because they do not show better results than direct method parameters in the literature [18]. Unlike the indirect methodologies, the new index RRR is not affected by changes in parenchymal stiffness, not based on the waveform and allows beam-angle corrections. Souza de Oliveira et al. showed that the renal segmental ratio (RSR), which was defined as the proximal RPSV divided by the segmental artery peak systolic velocity, was able to predict the presence of significant stenosis at the main renal artery. The RSR showed satisfactory results for sensitivity (83.3%) and low specificity (78.9%) for all renal units. This study analyzed intrarenal segmental velocities that were subjeted to more variable factors, particularly the elastic properties of the arterial vessel walls, changes in peripheral resistance within renal vascular circuits and changes in parenchymal stiffness [14]. In our current study, the RRR results for sensitivity and specificity were 97 and 96%, respectively. These data might be explained by the fact that we employed the RPSV in the extraparenchymal segment, which was not subjet to variability in the factors mentioned above [14].

Van der Hulst et al. found that the ratio between the maximum peak systolic velocity at the renal artery and the minimum value at the arcuate artery, analysed using an endovascular procedure, gave good results for RAS diagnosis (a sensitivity of 94% and a specificity of 100%). However, their study used an invasive approach and had a higher risk than the RRR proposed in our current research [19].

Our best cutoff value for RRR was 2.7 in accordance with an angiographic RAS >50%; however, we found an angiographic RAS >50% with an RRR >2.5. We did not find RAS when the RRR was <2, so we proposed this cutoff to be the normal value.

Body mass index (BMI) was calculated for each patient. The mean BMI was 30 (± 5) kg/m^2^. (Table 2). The 97% of our patients were successfully examined with color Doppler US, even in those with a high BMI.These favourable results might have been related to the strict bowel preparation and the fact that the examinations were performed by a trained specialist.

This index was assessed in all patients with only one exception. This patient had RAS at the end of the distal renal artery segment, so we could not take a distal velocity to compare with the RPSV at the RAS. We therefore used to replace the distal renal artery segment velocity, the measurement at non-stenotic distal branch of the main renal artery at the extraparenchymal level.

The main limitation of this study was that we evaluated only the main renal arteries because these vessels have a more important role at the renovascular disease and were able to undergo endovascular treatment. Neither we compared the color Doppler US with other diagnostic non-invasive diagnostic methods as computed tomographic, magnetic resonance angiography or captopril renography for the diagnosis of RAS. We did not considerate these diagnostic methods in this study because their high cost and not widespread availability of all of them may somehow avoid a routine use in clinical practice [22].

## Conclusion

The new RRR, with a cutoff value >2.7, shows significantly improved specificity compared with conventional direct method Doppler parameters (RPSV > 200 cm/s and RAR >3). Both RRR and RPSV show better sensitivity than RAR for RAS diagnosis. The results indicate that RRR and RPSV were the best criteria for RAS diagnosis. The RRR improves the diagnostic effectiveness of the color Doppler US.

## List of abbreviations

RPSV = renal peak systolic velocity

RAR = renal aortic ratio

RRR = renal-renal ratio

RAS = renal artery stenosis

Renal color Doppler ultrasound = color Doppler US

DSA = digital subtraction arteriography

ROC curve = receiver-operating characteristic curve

PW Doppler = pulsed wave Doppler

## Competing interests

The author(s) declare that they do not have competing interests.

## Authors' contributions

SC designed the study and carried out the sonographic evaluations.

HL, GF, SB, JO and JM performed literature research and prepared, edited and reviewed the manuscript.

EME and RR participated in the design and coordination of the study, and SC, HL, GF and SF guarantors of its integrity.

All authors read and approved the final manuscript.
